# Supplementary material for: RNA polymerase II is a polar roadblock to a progressing DNA fork
Source: Nat Commun. 2025 Sep 29;16:8631. doi: 10.1038/s41467-025-63662-1 (PMC12479915; doi:10.1038/s41467-025-63662-1)
Supplement: Supplementary file 1 — Supplementary Information [file 41467_2025_63662_MOESM1_ESM.pdf]

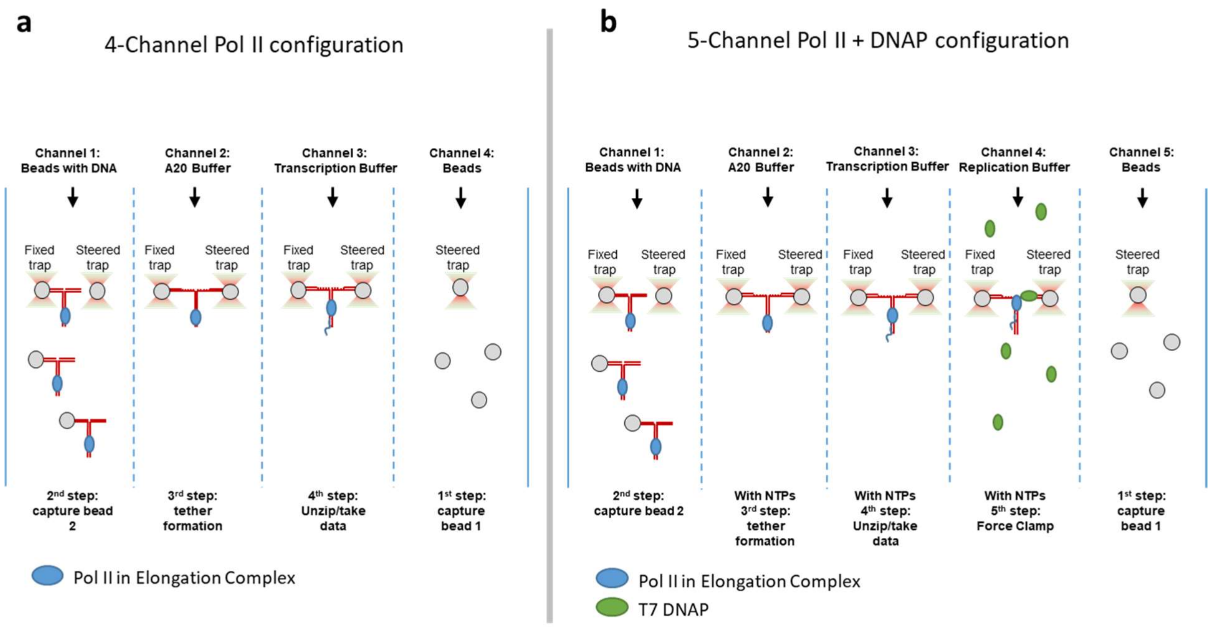

Supplementary Figure 1. The multi-channel microfluidic flow cells

a. The four-channel flow cell used for data collection for Figures 1, 2, 3, and 4. This flow cell, combined with a dual-trap optical tweezer, utilized laminar flow to partition different buffers, allowing for tether formation and movement into various experimental conditions. This allowed for transcription to only begin after a tether was formed and ready for data collection.

b. The five-channel flow cell used for data collection for Figure 5. This flow cell, combined with a dual-trap optical tweezer, utilized laminar flow to partition different buffers, allowing for tether formation and movement into various experimental conditions. This allowed for a single Pol II molecule to begin transcription and then be moved into a replication channel only after RNA-DNA hybrid formation.

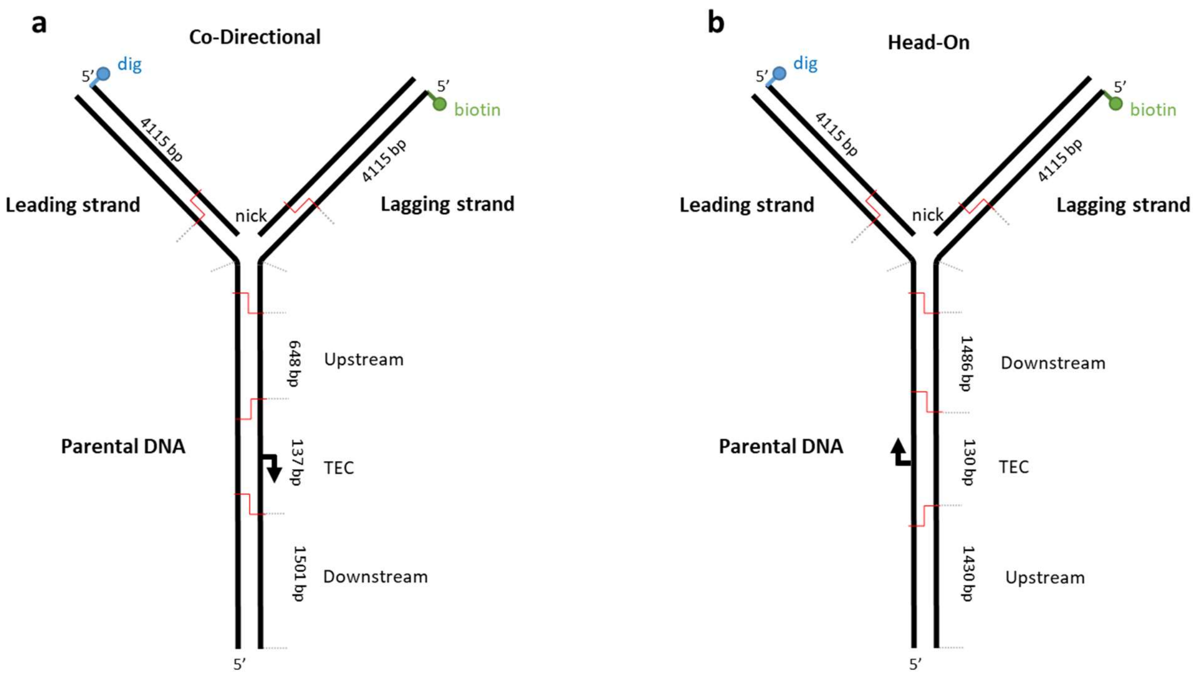

Supplementary Figure 2. The Y-shaped DNA substrates

a. The co-directional Y-shaped DNA substrate. The template consists of two labeled daughter strands to allow for tethering between the trapped beads. The daughter strands are connected to a three-way junction to resemble a replication fork. The parental strand is assembled from the fork as follows: upstream DNA, assembled Pol II TEC, and then downstream DNA, allowing for co-directional collision studies where Pol II moves away from the DNA fork.

b. The head-on Y-shaped DNA substrate. The template consists of two labeled daughter strands to allow for tethering between the trapped beads. The daughter strands are connected to a three-way junction to resemble a replication fork. The parental strand is assembled from the fork as follows: downstream DNA, assembled Pol II TEC, and then upstream DNA, allowing for head-on collision studies where Pol II moves towards the DNA fork.

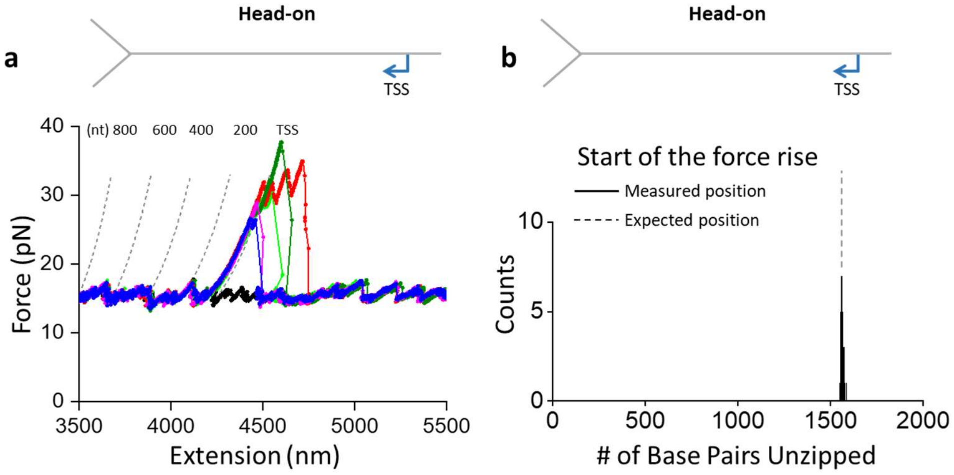

Supplementary Figure 3. Pol II paused at A20 in the head-on orientation.

a. Additional representative force-extension traces of the DNA fork unzipping through a paused Pol II at A20 in the head-on orientation. For each trace, the start of the force rise above the naked DNA baseline indicates the detection of a bound protein, and in this case, a bound Pol II. Using the start of this force rise, the initial position of the front edge of Pol II can be mapped along the DNA sequence.

b. The histogram for the detected position of the Pol II along the DNA sequence, as indicated by the number of base pairs unzipped. The measured position of Pol II's front edge is at 1562 ± 8 bp, close to the expected position of 1556 bp. This histogram shows that a Pol II population (N = 28) with a rather homogeneous starting position can have different disruption forces and sliding behaviors as shown in **a**.

Source data are provided as a Source Data file.

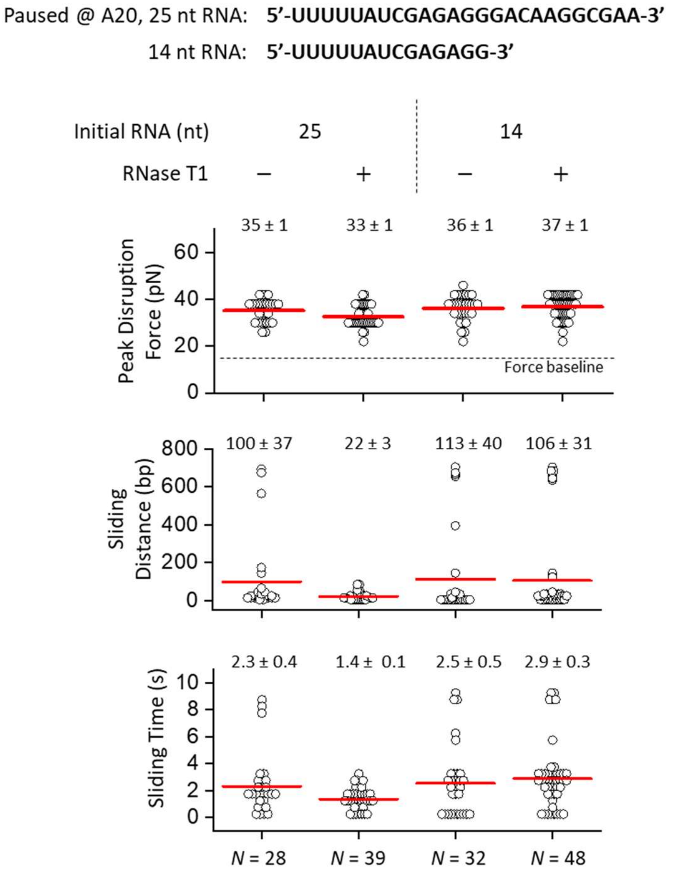

Supplementary Figure 4. Sliding distance and time of a head-on paused Pol II.

Figure 1 shows that a head-on Pol II paused at A20 (25 nt RNA) can resist unzipping by sliding along the DNA. For a sliding distance smaller than the transcript length, a possible sliding mode is backtracking, where the transcription bubble maintains full RNA-DNA hybrid within the bound Pol II. However, if the sliding distance exceeds the transcript size, the RNA-DNA hybrid can no longer be maintained within the transcript bubble. In this case, it is unclear how Pol II remains bound to the DNA. One possibility is for Pol II to assume an orientation akin to an open complex, which is known to be short-lived and prone to bubble collapse over a time scale of seconds[1]. We examined the sliding time of an A20-paused Pol II in the head-on orientation. The lifetime of this orientation is indeed short (a few seconds), similar to the lifetime of an open complex. However, the paused A20 Pol II data also suggests that long-distance sliding increases with RNA transcript size, as the presence of RNase T1 reduces the sliding distance. This indicates that the sliding configuration may not be a simple open complex, but rather one that also involves RNA, with its ability to slide likely dependent on the DNA sequence. Thus, this configuration is short-lived, but the exact nature of the orientation remains unclear.

To examine whether RNase T1 may impact the roadblock properties of Pol II beyond its known function of cutting the RNA, we conducted an additional head-on orientation experiment using the assembled elongation complex before any NTP chase. This complex contains 14 nt RNA, with 9 nt RNA complementary to the template strand and 5' UUUUU tail non-complementary to the template (see Methods). The U-tail can potentially stabilize the elongation complex via interactions with the RNA channel of Pol II[2], but it cannot be cut by RNase T1. We found that the disruption force and sliding distance remain the same in the absence and presence of RNase T1, indicating that RNase T1 does not impact any detectable properties of the elongation complex.

Source data are provided as a Source Data file.

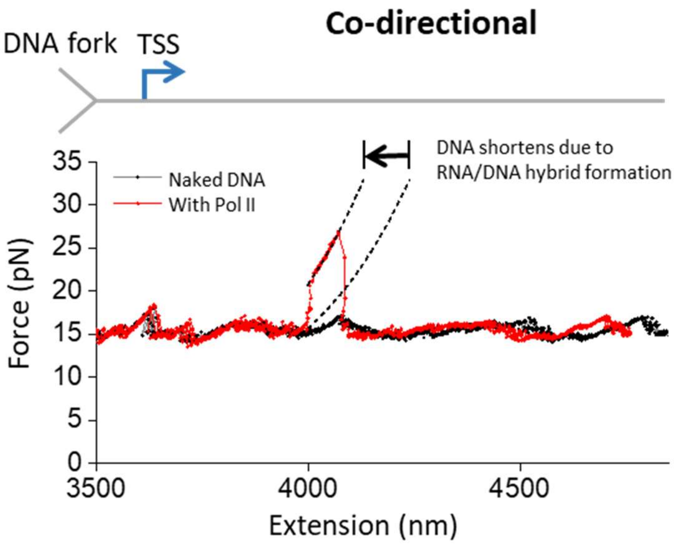

Supplementary Figure 5. Co-directional RNA-DNA hybrid extension shift in force peak

Representative trace showing the interaction of the DNA fork with an elongating Pol II elongation complex (EC) in the co-directional (CD) orientation. The force-extension data of DNA with Pol II bound are shown in red while those of naked DNA of the same sequence are shown in black. As shown in Figure 2a, immediately upon the unzipping fork encountering the bound Pol II from behind, the force-extension curve snapped back and followed a curve at a shorter extension until the disruption of the bound Pol II. The two dashed curves are used to show the initial Pol II position (right) and the Pol II position after RNA-DNA hybrid formation (left) before disruption of the protein. This snap-back behavior may be a result of RNA-DNA hybrid formation behind Pol II, with RNA-DNA hybrid formation on the leading strand shortening the overall DNA extension (Fig. 2b). The initial Pol II position is shown using a freely-jointed chain (FJC) model, with only ssDNA contributing; the shifted Pol II position is plotted using a combined FJC and worm-like chain (WLC) model due to having a hybrid component present. The black arrow indicates the amount of extension shortening. The TSS is illustrated in the cartoon above, with the blue arrow indicating the direction of movement of elongating Pol II.

Source data are provided as a Source Data file.

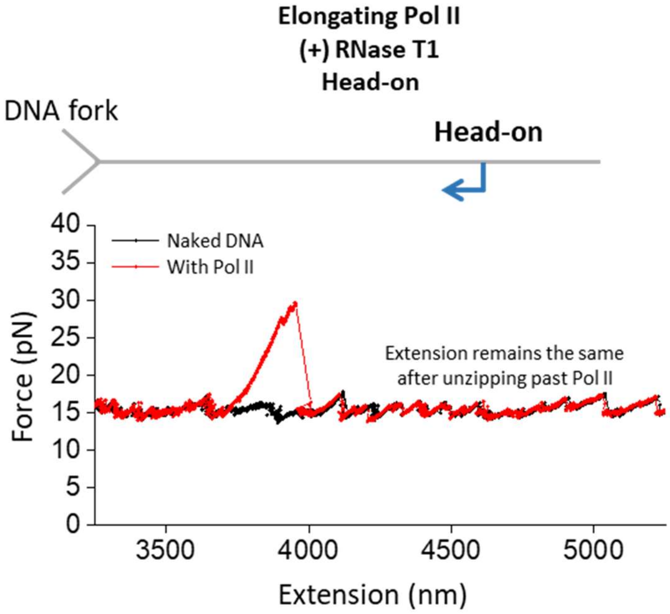

Supplementary Figure 6. Head-on RNase T1 example trace

Representative trace showing the interaction of the DNA fork with an elongating Pol II elongation complex (EC) in the head-on (HO) orientation in a (+) RNase T1 environment. The force-extension data of DNA with Pol II bound are shown in red while those of naked DNA of the same sequence are shown in black. No shift in extension is measured after disruption of Pol II or unzipping past the TSS, due to a lack of RNA-DNA hybrid formation as the majority of the RNA should have been digested by RNase T1. The TSS is illustrated in the cartoon above, with the blue arrow indicating the direction of elongating Pol II.

Source data are provided as a Source Data file.

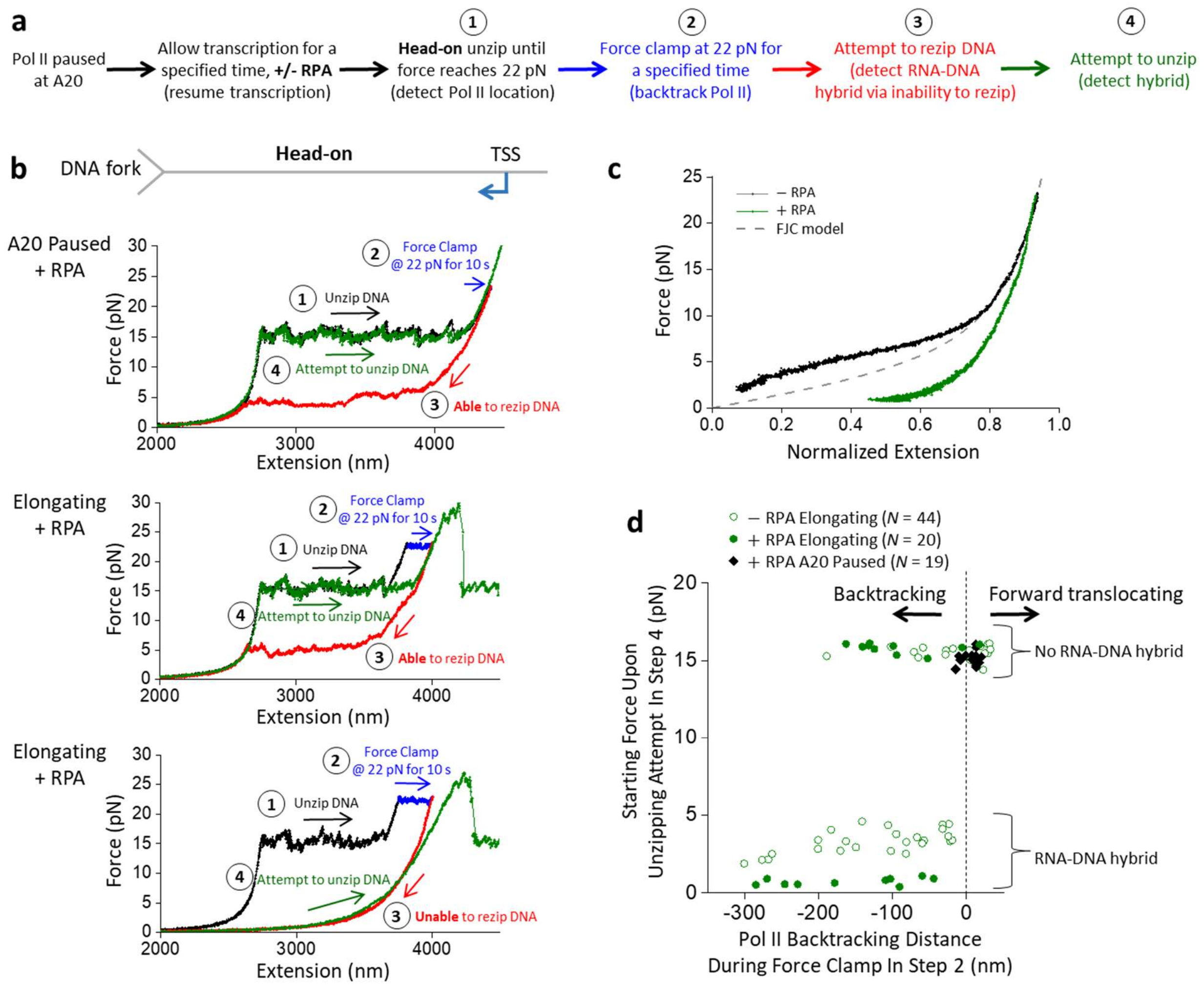

Supplementary Figure 7. RNA-DNA hybrid formation in front of Pol II in the presence of RPA.

The experiments shown in Figs. 1-5 are conducted in the absence of single-stranded binding protein, RPA. *In vivo*, RPA may bind to the lagging strand ahead of Pol II during a head-on collision with a replisome, and RPA binding may compete with the formation of an RNA-DNA hybrid. To examine this possibility, we carried out the experiments as outlined in Fig. 4a except in the presence of 5 nM RPA during transcription. This RPA concentration is more than an order of magnitude higher than the K_d of RPA to ssDNA[3].

a. Outline of the experimental steps to backtrack Pol II and determine if RNA-DNA hybrid formation occurred. These steps are identical to those shown in Fig. 4a.

b. Representative force-extension traces in the head-on orientation in the presence of RPA. The top trace is taken with Pol II paused at A20, a condition that limits RNA-DNA hybrid formation

due to short RNA. Under this RPA concentration, RPA bound on ssDNA can effectively resist DNA rezipping: without RPA, DNA can be rezipped at around 15 pN; however, with RPA, rezipping is prevented until the force is lowered to around 5 pN, when rezipping becomes energetically favorable, and the DNA is subsequently fully rezipped. In step 4, the ability to unzip the DNA further verifies that the DNA has been fully rezipped. These observations provide evidence that RPA effectively binds to the ssDNA, and bound RPA proteins block DNA rezipping until the force is so low that rezipping becomes energetically favorable. The middle trace is taken with an elongating Pol II. For this trace, the DNA is fully rezipped at the end of step 3, indicating the lack of RNA-DNA hybrid formation. The bottom trace is also taken with an elongating Pol II. For this trace, the DNA cannot be rezipped in step 3, even when the force is lowered below 5 pN, consistent with RNA-DNA hybrid preventing the final rezipping. Thus, RPA cannot block the final rezipping, and the blockage of the final rezipping requires RNA-DNA hybrid formation. This is further verified in step 4, which shows a low force (~ 0.4 pN) near 2750 nm at the start of the expected unzipping. These data demonstrate that a low starting force upon the unzipping attempt in step 4 can serve as an accurate indicator of the RNA-DNA hybrid formation.

c. RPA binding inhibits the formation of secondary structures of ssDNA. Shown are example traces of force-extension curves, with extension normalized against the ssDNA contour length. In the absence of RPA, the force-extension of ssDNA is well-described by the freely-jointed-chain (FJC) model using a persistence length of 0.85 nm and a stretch modulus of 504 pN[4] at forces > 10 pN. Below this force, the measured force of ssDNA is greater than the FJC model prediction, consistent with ssDNA forming secondary structures in the low-force regime. In the presence RPA, the measured force is significantly reduced in the low-force region, consistent with RPA binding limiting the formation of secondary structures in ssDNA.

d. Detection of RNA-DNA hybrid formation via the starting force upon the unzipping attempt in step 4. A force around 15 pN indicates a lack of formation of a hybrid. A force value significantly lower than this indicates hybrid formation. For comparison, the corresponding elongating Pol II data in the absence of RPA are also plotted. This plot shows that an RNA-DNA hybrid can still form rather effectively on the lagging strand in front of an elongating Pol II in the presence of RPA.

Source data are provided as a Source Data file.

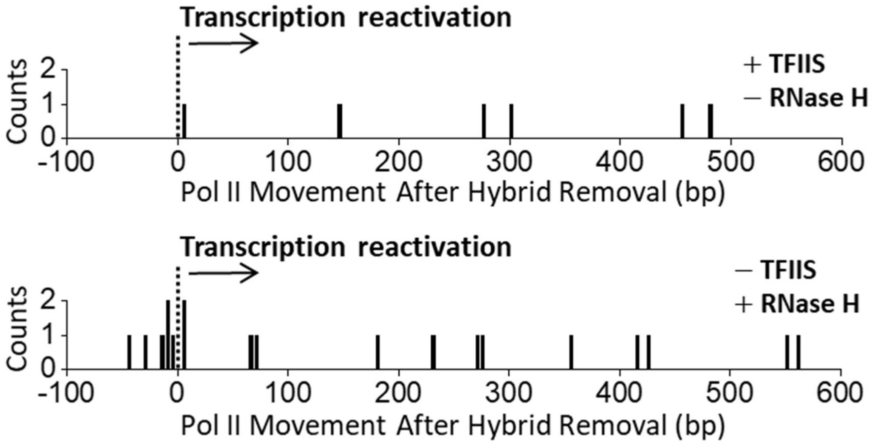

Supplementary Figure 8. Transcription resumption after RNA-DNA hybrid removal

Pol II movement as assessed in step 4 for all traces that formed an RNA-DNA hybrid after Pol II backtracking and had subsequent hybrid removal. (+) TFIIS (*N* = 6) and (+) RNase H (*N* = 19).

Source data are provided as a Source Data file.

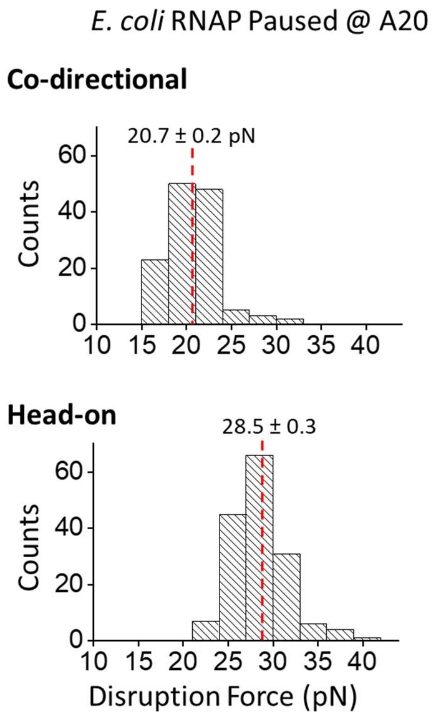

Supplementary Figure 9. *E. coli* RNAP is a similar polar roadblock as Pol II to a progressing DNA fork.

We have previously investigated the disruption of *E. coli* RNA polymerase (RNAP) using the DNA unzipping mapper method in both co-directional and head-on orientations[5]. We re-analyzed the data and plotted the disruption force. We found that *E. coli* RNAP exhibits a similar polarity to the Pol II roadblock at a DNA fork, displaying a greater disruption force in the head-on orientation than in the co-directional orientation. This suggests that the polarity may be the same for the transcription elongation complex in both prokaryotes and eukaryotes.

Source data are provided as a Source Data file.

Supplementary Table 1. Primer and oligo sequences.

**Oligo Name**	**Sequence (5' → 3')**
RNA 14	UUUUU<u>AUCGAGAGG</u> (Underlined sequence complementary to the gap)
ARP 77	/5Phos/TCAGCCCTATAGGATACTTACAGCCATCGAGAGGGACAAGGCGAATACCCATCCCAATCGGCCTGCTGGTGACACC
US NTS	/5Phos/GTCCTAATTCGAGCTCGGTACCCGGGGATCC
DS NTS	/5Phos/TCAGCGGATCCTCTAGAGTCCTTCAGCGAT
TS	CTGAAGGACTCTAGAGGATCCGCTGAGGTGTCACCAGCAGGCCGATT
0.65/1.4 kb CD/HO upstream PCR F	GCTCCTGTCGTTGAGGACCC
0.65 kb CD upstream PCR R	GCTTACAGACACCTAGTGACCG
1.4 kb HO upstream PCR R	CTGCTAATCCTGTTACCAGCTACTGC
1.5 HO/CD Downstream PCR F	AATACTGTTCACCAACGATCTGGATCACG
1.5 HO/CD Downstream PCR R	GGGACACACACGCCAGCTACTG
Daughter Strand PCR F	CGCGTTTCGGTGATGACGGTGA
Leading Strand PCR R_Bio	/5BiosG/TACCGATGAAACGAGAGAGGATGC
Lagging Strand PCR R_Dig	/5DiGN/TACCGATGAAACGAGAGAGGATGC
Upper Leading Strand	/5Phos/GGGACAGACGCTGTCCGCGCCAGTGCAGAATAAGGAGTCATTCGTGGGGTGGAC
Upper Leading Strand_invdT	/5Phos/GGGACAGACGCTGTCCGCGCCAGTGCAGAATAAGGAGTCATTCGTGGGG/3invdT/

Lower Leading Strand	/5Phos/ACCCAGATGCGTGTGCGTAGAGCGGACCGCGTCCACCCCACGAATGACTCCTTATTCTGCACTGGCGCGGACAGCGTCTG
Upper Lagging Strand	CAGCGCCAGACTGGGGGCGTCCTGCAGAAGGCTCCCACGACGACACCGAC
Lower Lagging Strand	/5Phos/GGGAGTCGGTGTCGTCGTGGGAGCCTTCTGCAGGACGCCCCCAGTCTGGCGCTGGCGGTCCGCTCTACGCACACGCATCTGGGTCTA

Supplementary References

1. Darzacq, X. *et al.* In vivo dynamics of RNA polymerase II transcription. *Nat. Struct. Mol. Biol.* **14**, 796–806 (2007).
2. Palangat, M. *et al.* Efficient reconstitution of transcription elongation complexes for single-molecule studies of eukaryotic RNA polymerase II. *Transcription* **3**, 146–153 (2012).
3. Kim, C., Snyder ,Richard O. & and Wold, M. S. Binding Properties of Replication Protein A from Human and Yeast Cells. *Mol. Cell. Biol.* **12**, 3050–3059 (1992).
4. Killian, J. L., Inman, J. T. & Wang, M. D. High-Performance Image-Based Measurements of Biological Forces and Interactions in a Dual Optical Trap. *ACS Nano* **12**, 11963–11974 (2018).
5. Le, T. T. *et al.* Mfd Dynamically Regulates Transcription via a Release and Catch-Up Mechanism. *Cell* **172**, 344-357.e15 (2018).
