# Supplementary material for: RNA polymerase II is a polar roadblock to a progressing DNA fork
Source: Nat Commun. 2025 Sep 29;16:8631. doi: 10.1038/s41467-025-63662-1 (PMC12479915; doi:10.1038/s41467-025-63662-1)
Supplement: Supplementary file 2 — Reporting Summary [file 41467_2025_63662_MOESM2_ESM.pdf]

nature portfolio

Corresponding author(s): Michelle D. Wang
Last updated by author(s): Aug 11, 2025

Reporting Summary

Nature Portfolio wishes to improve the reproducibility of the work that we publish. This form provides structure for consistency and transparency in reporting. For further information on Nature Portfolio policies, see our Editorial Policies and the Editorial Policy Checklist.

Statistics

For all statistical analyses, confirm that the following items are present in the figure legend, table legend, main text, or Methods section.

n/a	Confirmed	
☐	☒	The exact sample size (*n*) for each experimental group/condition, given as a discrete number and unit of measurement
☐	☒	A statement on whether measurements were taken from distinct samples or whether the same sample was measured repeatedly
☐	☒	The statistical test(s) used AND whether they are one- or two-sided<br>*Only common tests should be described solely by name; describe more complex techniques in the Methods section.*
☒	☐	A description of all covariates tested
☒	☐	A description of any assumptions or corrections, such as tests of normality and adjustment for multiple comparisons
☐	☒	A full description of the statistical parameters including central tendency (e.g. means) or other basic estimates (e.g. regression coefficient) AND variation (e.g. standard deviation) or associated estimates of uncertainty (e.g. confidence intervals)
☐	☒	For null hypothesis testing, the test statistic (e.g. *F*, *t*, *r*) with confidence intervals, effect sizes, degrees of freedom and *P* value noted<br>*Give P values as exact values whenever suitable.*
☒	☐	For Bayesian analysis, information on the choice of priors and Markov chain Monte Carlo settings
☒	☐	For hierarchical and complex designs, identification of the appropriate level for tests and full reporting of outcomes
☒	☐	Estimates of effect sizes (e.g. Cohen's *d*, Pearson's *r*), indicating how they were calculated

Our web collection on statistics for biologists contains articles on many of the points above.

Software and code

Policy information about availability of computer code

Data collection	LabView, custom optical tweezers software (LabVIEW 2020)
Data analysis	MATLAB Version: 24.2.0.2863752 (R2024b) (https://github.com/WangLabCornell/Kay_et_al)

For manuscripts utilizing custom algorithms or software that are central to the research but not yet described in published literature, software must be made available to editors and reviewers. We strongly encourage code deposition in a community repository (e.g. GitHub). See the Nature Portfolio guidelines for submitting code & software for further information.

Data

Policy information about availability of data

All manuscripts must include a data availability statement. This statement should provide the following information, where applicable:

- Accession codes, unique identifiers, or web links for publicly available datasets
- A description of any restrictions on data availability
- For clinical datasets or third party data, please ensure that the statement adheres to our policy

All data are available in the main text or the supplementary materials. Source Data are provided with this paper.

Research involving human participants, their data, or biological material

Policy information about studies with human participants or human data. See also policy information about sex, gender (identity/presentation), and sexual orientation and race, ethnicity and racism.

Reporting on sex and gender	NA
Reporting on race, ethnicity, or other socially relevant groupings	NA
Population characteristics	NA
Recruitment	NA
Ethics oversight	NA

Note that full information on the approval of the study protocol must also be provided in the manuscript.

Field-specific reporting

Please select the one below that is the best fit for your research. If you are not sure, read the appropriate sections before making your selection.

☒ Life sciences ☐ Behavioural & social sciences ☐ Ecological, evolutionary & environmental sciences

For a reference copy of the document with all sections, see nature.com/documents/nr-reporting-summary-flat.pdf

Life sciences study design

All studies must disclose on these points even when the disclosure is negative.

Sample size	No formal sample size calculation was performed. Each data point corresponds to a unique DNA molecule. The sample size was based on prior experience with single-molecule measurements and established practices in the field. The number of replicates for each experimental condition was between N = 8 and 60. Detailed statistical information, including the number of traces, mean values, and standard error of the mean (SEM), is provided in the manuscript text, figures, and figure legends.
Data exclusions	Data was excluded from the analysis if there were DNA tethering issues (e.g., DNA broke during measurement), no bound RNA Polymerase II elongation complex was present on the DNA, or the RNA Polymerase II molecule was inactive. Only tethers with active RNA Pol II were analyzed.
Replication	All experiments described in the manuscript were reproducible. Data for each condition were taken over multiple days (at least two independent days) to ensure reproducibility. For each condition, data were collected from multiple unique DNA molecules, with the number of replicates ranging from N = 8 to 60.
Randomization	Randomization was not relevant to this study, all experiments were carried out using the same experimental procedures.
Blinding	Blinding was not relevant to this study. All data collection and analysis were performed using standardized procedures that were applied uniformly across all samples and experimental conditions. All data collection and analysis were based on objective, quantitative measurements obtained from single-molecule optical tweezers experiments.

Reporting for specific materials, systems and methods

We require information from authors about some types of materials, experimental systems and methods used in many studies. Here, indicate whether each material, system or method listed is relevant to your study. If you are not sure if a list item applies to your research, read the appropriate section before selecting a response.

Materials & experimental systems

n/a	Involved in the study	
☐	☒	Antibodies
☒	☐	Eukaryotic cell lines
☒	☐	Palaeontology and archaeology
☒	☐	Animals and other organisms
☒	☐	Clinical data
☒	☐	Dual use research of concern
☒	☐	Plants

Methods

n/a	Involved in the study	
☒	☐	ChIP-seq
☒	☐	Flow cytometry
☒	☐	MRI-based neuroimaging

Antibodies

Antibodies used

Sheep anti-digoxigenin, Roche, 11333089001, Lot# 60789300 and 67442300

Validation

Anti-digoxigenin was used to tether DNA to a polystyrene bead. It was validated by the observation of DNA labeled with digoxigenin tethered to the bead. The specificity and quality of the anti-body does not affect the outcome of the single-molecule study. No additional data are necessary to validate the antibody.

Plants

Seed stocks

Report on the source of all seed stocks or other plant material used. If applicable, state the seed stock centre and catalogue number. If plant specimens were collected from the field, describe the collection location, date and sampling procedures.

Novel plant genotypes

Describe the methods by which all novel plant genotypes were produced. This includes those generated by transgenic approaches, gene editing, chemical/radiation-based mutagenesis and hybridization. For transgenic lines, describe the transformation method, the number of independent lines analyzed and the generation upon which experiments were performed. For gene-edited lines, describe the editor used, the endogenous sequence targeted for editing, the targeting guide RNA sequence (if applicable) and how the editor was applied.

Authentication

Describe any authentication procedures for each seed stock used or novel genotype generated. Describe any experiments used to assess the effect of a mutation and, where applicable, how potential secondary effects (e.g. second site T-DNA insertions, mosiacism, off-target gene editing) were examined.
